# Spatial distribution of insulin-like growth factor binding protein-2 following hypoxic-ischemic injury

**DOI:** 10.1186/1471-2202-14-158

**Published:** 2013-12-21

**Authors:** Lauren Fletcher, Elif Isgor, Shane Sprague, Lindsey H Williams, Betty B Alajajian, David F Jimenez, Murat Digicaylioglu

**Affiliations:** 1 Department of Neurosurgery, University of Texas Health Science Center at San Antonio, San Antonio, TX 78229, USA; 2 School of Medicine, University of Texas Health Science Center at San Antonio, San Antonio, TX 78229, USA; 3 Department of Physiology, University of Texas Health Science Center at San Antonio, San Antonio, TX 78229, USA

**Keywords:** Stroke, Ischemia, Neuroprotection, Insulin-like growth factor binding protein-2, Insulin-like growth factor-I, Intranasal administration

## Abstract

**Background:**

Insulin-like growth factor binding protein-2 (IGFBP-2) regulates the bioavailability, transportation, and localization of insulin-like growth factor-I (IGF-I), an effective neuroprotectant in animal stroke models especially when administered intranasally. Therefore, determining IGFBP-2′s endogenous distribution in the normal and ischemic brain is essential in maximizing the neuroprotective potential of the intranasal IGF-I treatment approach. However, current data on IGFBP-2 is limited to mRNA and in situ hybridization studies. The purpose of this study was to determine if there are any changes in IGFBP-2 protein levels and distribution in ischemic brain and also to determine if IGFBPs play a role in the transportation of intranasally administered IGF-I into the brain.

**Results:**

Using an *in vitro* approach, we show that ischemia causes changes in the distribution of IGFBP-2 in primary cortical neurons and astrocytes. In addition, we show using the transient middle cerebral artery occlusion (MCAO) model in mice that there is a significant increase in IGFBP-2 levels in the stroke penumbra and core after 72 h. This correlated with an overall increase in IGF-I after stroke, with the highest levels of IGF-I in the stroke core after 72 h. Brain sections from stroke mice indicate that neurons and astrocytes located in the penumbra both have increased expression of IGFBP-2, however, IGFBP-2 was not detected in microglia. We used binding competition studies to show that intranasally administered exogenous IGF-I uptake into the brain is not receptor mediated and is likely facilitated by IGFBPs.

**Conclusions:**

The change in protein levels indicates that IGFBP-2 plays an IGF-I-dependent and -independent role in the brain’s acute (neuroprotection) and chronic (tissue remodeling) response to hypoxic-ischemic injury. Competition studies indicate that IGFBPs may have a role in rapid transportation of exogenous IGF-I from the nasal tissue to the site of injury.

## Background

Stroke is among the leading causes of neurological disability and has a devastating emotional and financial burden on stroke survivors and their families [1]. The only treatment currently available is thrombolysis with tissue plasminogen activator (tPA). However, due to the limited time window for treatment (<4.5 h) and the rigorous inclusion criteria, less then 5% of stroke patients are eligible for tPA treatment [2]. Unfortunately, there are no other efficient therapies to ameliorate the resulting neurodegeneration caused be cerebral ischemia. Recent studies have shown that intranasal administration of insulin-like growth factor-I (IGF-I) is neuroprotective in animal stroke models [3-5]. Other administration methods that deliver drugs systemically depend on the breakdown of the blood–brain barrier (BBB), however, this is not a predictable event in the ischemic brain [6,7]. Therefore, it is unreasonable to rely on the breakdown of the BBB to administer intravascular drugs as neurological therapeutics. In contrast, intranasal administration circumvents the BBB and is capable of directly transporting drugs [8] and even cells [9] into the central nervous system (CNS) via transcellular pathways [10], among them IGF-I [4]. However, the underlying mechanism for intranasal IGF-I uptake from the nasal cavity and its delivery to the injury site are still unknown [11].

A strong candidate suggested for the mediation of exogenous IGF-I is the insulin-like growth factor binding protein (IGFBP) family [12]. IGFBPs are highly conserved among mammalian species and are essential to IGF-I’s normal function as they are crucial regulators of IGF-I bioavailability, transportation and localization [13]. Furthermore, IGFBPs are present in both human [14] and rat [15] olfactory epithelia and olfactory bulb making it a likely candidate for the uptake of IGF-I from the nasal cavity and its transportation across the olfactory bulb to the injury site. Studies that use des-IGF-I, an analogue of IGF-I that has a weak affinity for IGFBPs, show that administration of des-IGF-I into the lateral cerebral ventricle of rats following hypoxic-ischemic injury did not decrease neuronal loss, whereas IGF-I significantly reduced neuronal loss when compared to the vehicle treated group [16]. This suggests that IGF-I requires IGFBPs to carry out its neuroprotective functions.

Among the IGFBPs present in the CNS, IGFBP-2 is essential in elucidating the mechanism behind IGF-I delivery. Even though the overall IGFBP-2 levels decrease after initial stages of development, it remains to be the most abundant IGFBP in the CNS [13]. Interestingly, IGF-I has high binding affinity for IGFBP-2, but this affinity greatly decreases when IGFBP-2 binds to cell surface proteins [17]. This decrease in affinity causes IGFBP-2 to release IGF-I in close proximity of IGF-I receptors (IGF-IRs) on the cellular surface. This interaction is thought to play a significant role in IGF-I localization to its receptor [18] and could also play a role in the initiation of the anti-apoptotic signaling cascade [19].

Furthermore, high levels of IGFBP-2 mRNA have been detected in major sites of IGF-I production in the adult rodent brain, such as the olfactory bulb [20]. It has also been shown that IGFBP-2 mRNA levels increase after hypoxic-ischemic injury to the brain [21], and that IGFBP-2 mRNA co-localizes with endogenous IGF-I at the injury site [22,23].

Despite data suggesting a role for IGFBP-2 in endogenous and exogenous IGF-I triggered neuroprotection, studies on IGFBP-2 currently have been limited to mRNA levels and in situ hybridization, with no indication of the actual protein levels and distribution. Therefore, the objective of this study was to determine how ischemic injury may effect IGFBP-2 protein levels in the mouse brain, particularly in the stroke penumbra, the main target of neuroprotective treatments [24]. Animal studies have shown that the effective treatment window for stroke is 3–6 h, yet they have also shown that later time points contribute to recovery via neurogenesis, angiogenesis and overall tissue repair [25]. However, the majority of stroke patients do not arrive at the hospital within the treatment window, and most clinical trials continue with the neuroprotective treatment they are analyzing for a few days after the initial administration [26]. These factors make it essential to establish the distribution and concentration of any neuroprotectant and its potential carriers past the acute phase. In the current study we chose to explore IGFBP-2 expression and distribution in the brain in the chronic phases of stroke [27], which would help elucidate if there is potential for further protection/repair of the neuron population and remodeling of the penumbra and core. Since intranasal administration is shown to be the most effective route for IGF-I mediated neuroprotection, we have also analyzed the olfactory epithelia and the olfactory bulb for IGFBP-2 levels and investigated the role of IGFBPs in transportation of intranasally administered IGF-I. Our results indicate that IGFBP-2 and IGF-I distribution drastically changes after hypoxic-ischemic injury and transportation of IGF-I from the nasal cavity to the brain is likely mediated by IGFBPs, and not the IGF-IR.

## Results

### *IGFBP-2* in ischemic cortical neurons and astrocytes

**Figure 1:**
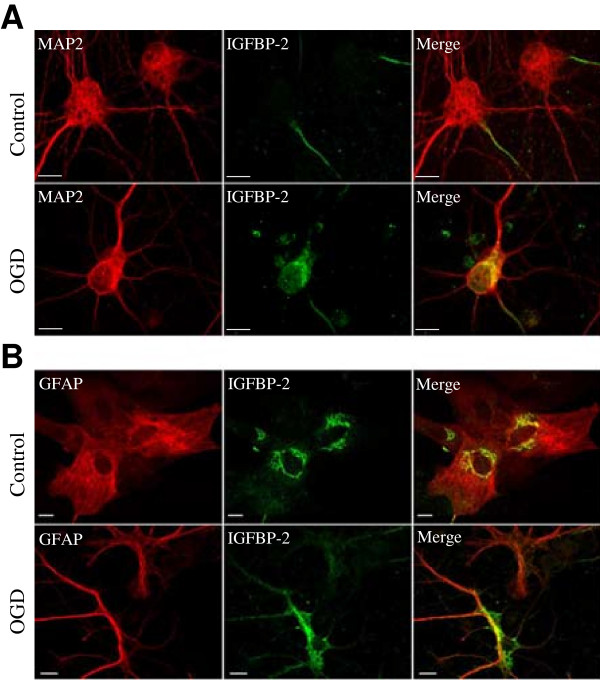
**IGFBP-2 in ischemic cerebrocortical neurons and astrocytes.** Primary neuron or astrocyte cultures were subjected to 1 h of oxygen-glucose deprivation (OGD) followed by 24 h of re-oxygenation. **(A)** Neurons are co-labeled with anti-MAP2 and anti-IGFBP-2. **(B)** Astrocytes are co-labeled with anti-GFAP and anti-IGFBP-2. Bar represents 10 μm.

First, we determined how ischemic conditions could affect IGFBP-2 *in vitro*. Primary neuron or astrocyte cultures were subjected to 1 h of oxygen-glucose deprivation (OGD) followed by 24 h of re-oxygenation to mimic ischemic stroke. The cells were then fixed and co-labeled with IGFBP-2 and/or microtubule-associated protein 2 (MAP2) or glial fibrillary acidic protein (GFAP) antibodies. Interestingly, under control conditions, the neurons expressed a small amount of IGFBP-2 that appeared to be only in the cells extensions (Figure 1A). After OGD, IGFBP-2 was seen throughout the cell body. In astrocytes under control conditions, IGFBP-2 immunoreactivity was seen mainly around the nucleus (Figure 1B). However, this changes in reactive astrocytes after OGD, in which IGFBP-2 is expressed throughout the entire cell body.

### IGFBP-2 and IGF-I in mouse brain

**Figure 2:**
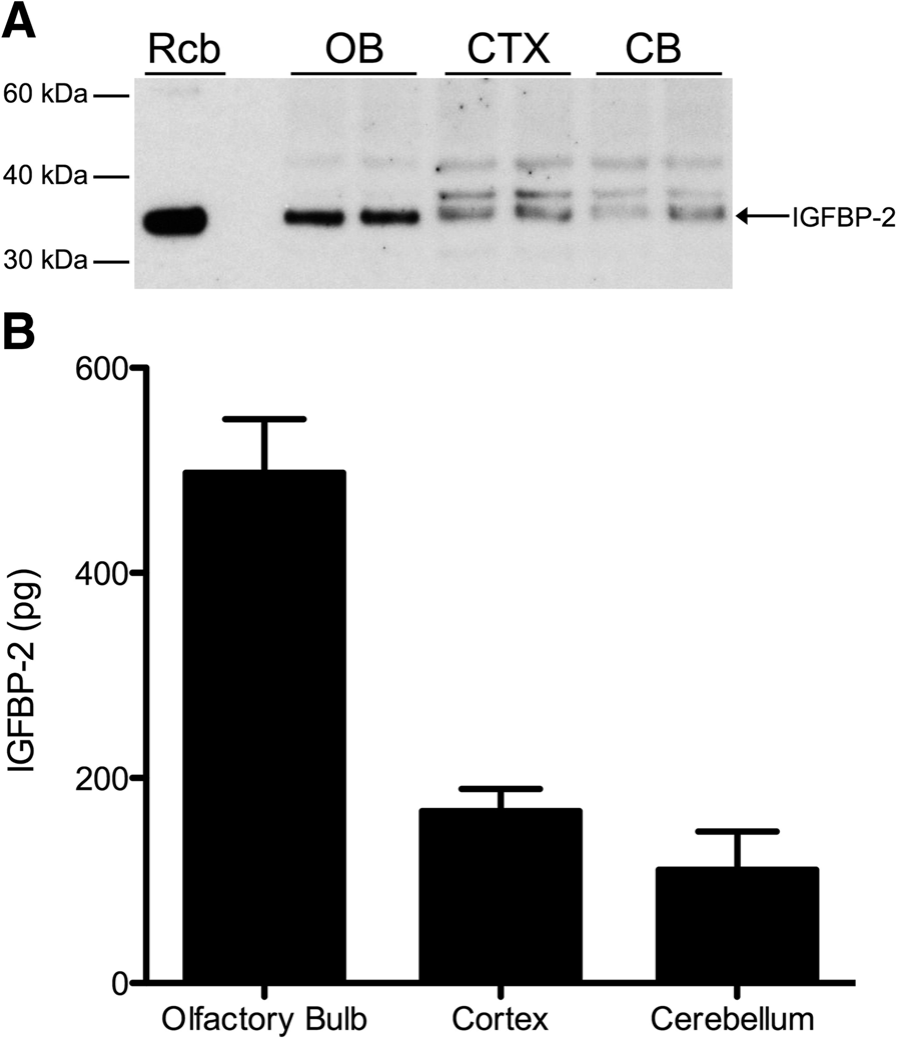
**IGFBP-2 protein in adult mouse brain.** IGFBP-2 is detectable in the olfactory bulb, cortex and cerebellum of adult mice. The olfactory bulb has the highest levels of IGFBP-2 compared to the rest of the tissue. **(A)** Western blot of IGFBP-2 in brain lysates. (Rcb - recombinant protein, OB - olfactory blulb, CTX - cortex, CB - cerebellum). **(B)** IGFBP-2 ELISA using brain lysates. Results are mean ± S.D. n = 5; *p < 0.001 by ANOVA, olfactory bulb versus cortex or cerebellum.

**Figure 3:**
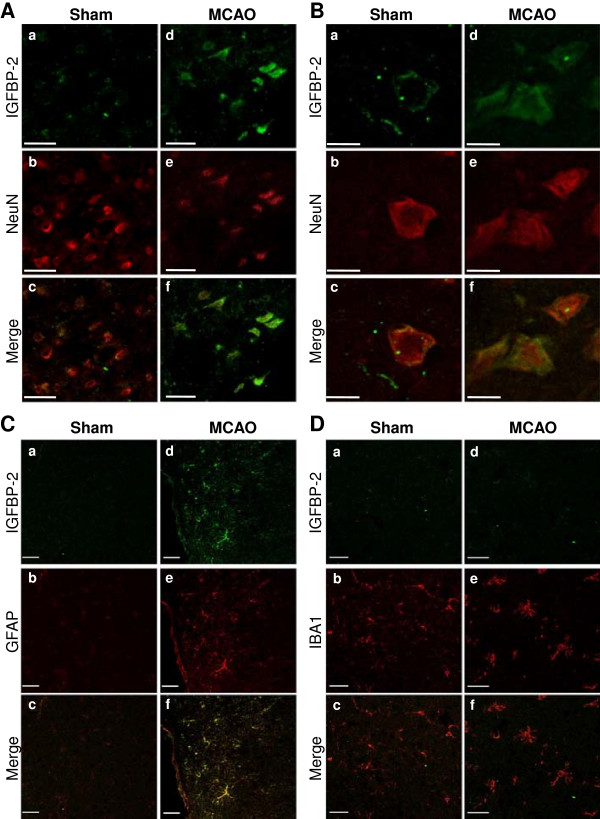
**IGFBP-2 Expression Following Hypoxic-Ischemic Injury.** Brain sections of mice from either the Sham group or the MCAO group were used to visualize IGFBP-2 in neurons, astrocytes and microglia. All images were taken of cells in the cortex that formed the penumbra. **(A, B)** Neurons are co-labeled with anti-NeuN and anti-IGFBP-2. **(C)** Astrocytes are co-labeled with anti-GFAP and anti-IGFBP-2. **(D)** Microglia are co-labeled with anti-Iba-1 and anti-IGFBP-2. Bars represent 20 μm.

**Figure 4:**
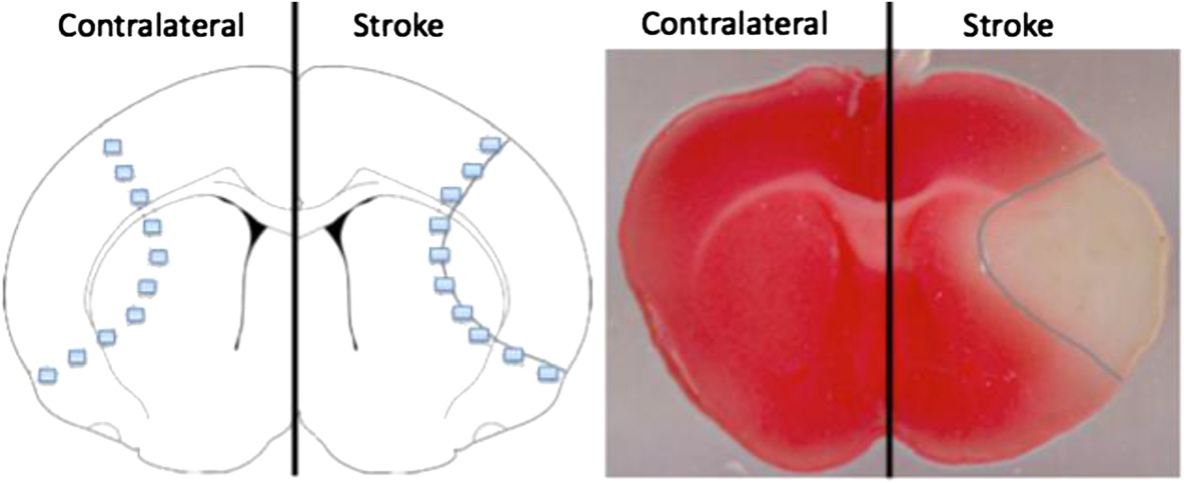
**Visualization of the stroke core and penumbra.** The stroke core is defined as the areas where perfusion is completely absent during occlusion, resulting in dead tissue. The stroke penumbra on the other hand receives limited perfusion during occlusion and still contains viable cells that are the main target of neuroprotectants. The drawing (left) shows how tissue collection was carried out. The squares on the drawing indicate the areas for penumbra collections (which correspond to the pink rim outlined in blue in the TTC image); the area inside the squares is the area used for stroke core collections (corresponding to the white area in the TTC image).

**Figure 5:**
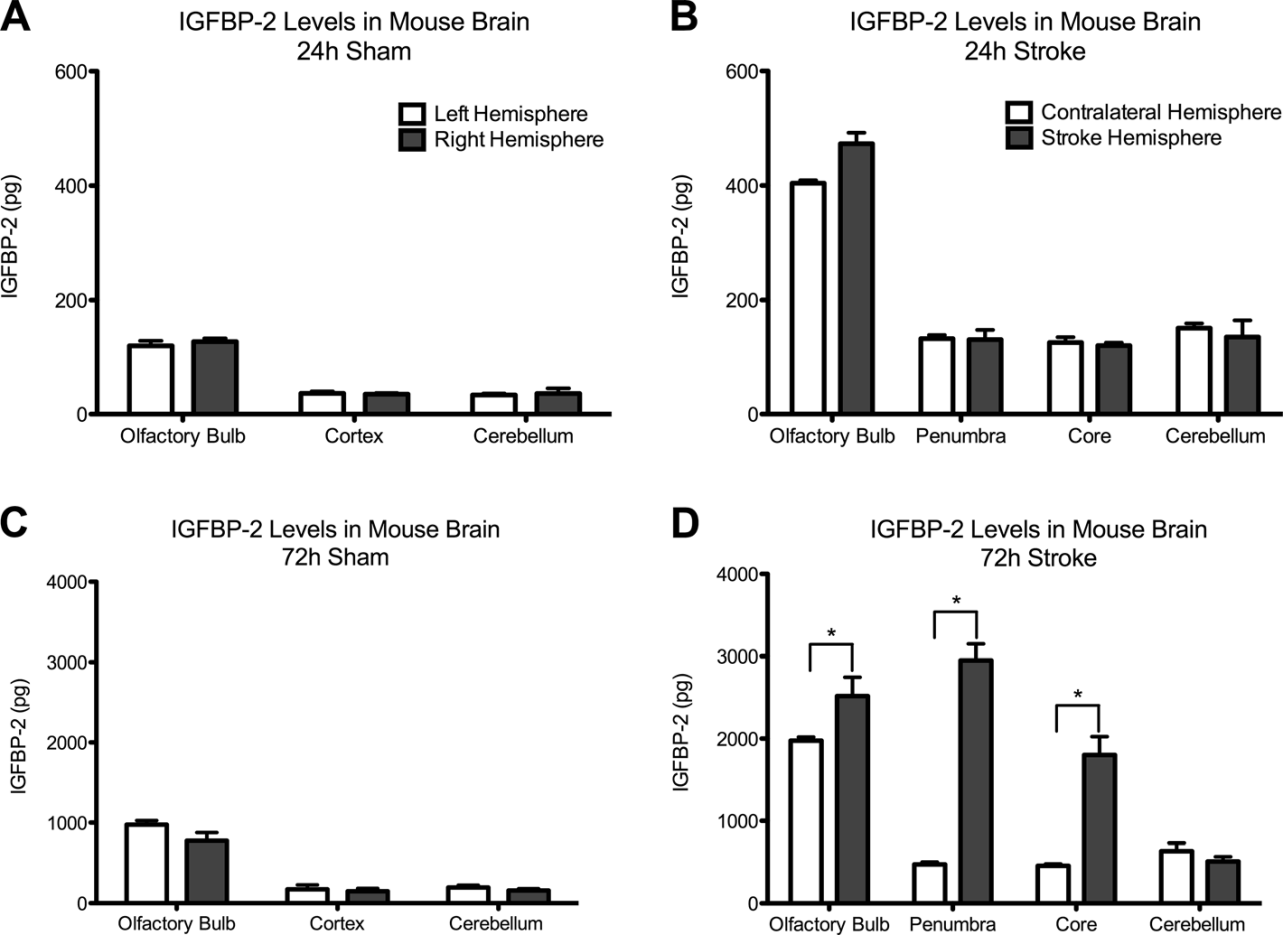
**IGFBP-2 protein levels following hypoxic-lschemic injury.** IGFBP-2 levels of sham animals are not significantly different between the left and the right hemispheres for the brain regions analyzed **(A, C)**. For the brain regions analyzed, IGFBP-2 levels are not significantly different between the contralateral and stroke hemispheres 24 h post-stroke **(B)**. IGFBP-2 levels on the stroke hemisphere increase significantly (*) in the penumbra (5-fold) and core (3-fold) when compared to the contralateral hemisphere 72 h post-stroke **(D)**. For statistical analysis, for each brain region, IGFBP-2 levels of the two hemispheres were compared. (*) on the graphs denotes the brain regions where the statistical analysis revealed a significant difference between the stroke and the contralateral hemisphere. Results are mean ± S.D. n = 5; *p < 0.05 by ANOVA contralateral hemisphere vs. stroke hemisphere.

**Figure 6:**
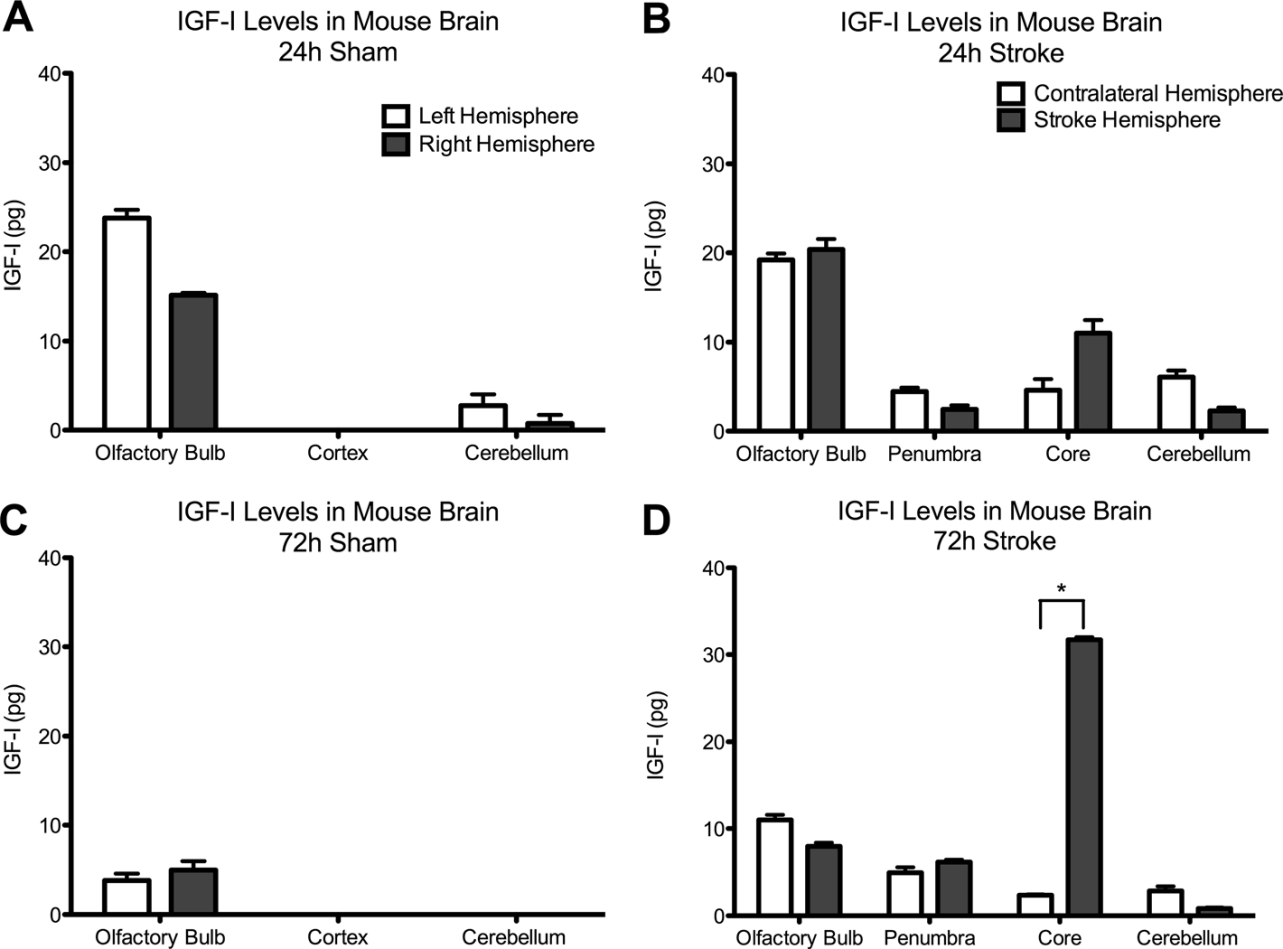
**IGF-I Levels Following Hypoxic-Ischemic Injury.** IGF-I levels of sham animals are not significantly different between the left and the right hemispheres for the brain regions analyzed **(A, C)**. IGF-I is detectable in the olfactory bulb, but not the cortex (24 h, 72 h) and cerebellum (72 h) of the sham animals **(A, C)**. IGF-I levels are highest in the olfactory bulb and detectable in the penumbra and core 24 h post-stroke **(B)**. At 72 h IGF-I levels are significantly higher in the core region (†) when compared to the other brain regions **(D)**. For statistical analysis, for each brain region, IGF-I levels of the two hemispheres were compared. (*) on the graphs denotes the brain regions where the statistical analysis revealed a significant difference between the stroke and the contralateral hemisphere. Results are mean ± S.D. n = 5; *p < 0.05 by ANOVA contralateral hemisphere vs. stroke hemisphere and †*p* < 0.05 by ANOVA core versus olfactory bulb, penumbra, cerebellum.

We characterized IGFBP-2 in the olfactory bulb, cortex and cerebellum in control mice by western blot and ELISA (Figure 2). IGFBP-2 protein was found to be most abundant in the olfactory bulb and was present in cortex and cerebellum in control animals. Next, we wanted to document the change in IGFBP-2 protein levels following hypoxic-ischemic injury. First, we explored the expression of IGFBP-2 in neurons, astrocytes and microglia in brain sections of mice that underwent 1 h of transient middle cerebral artery occlusion (MCAO). Images were taken of cells in the cortex that formed the penumbra. Compared to sham animals, both neurons and astrocytes show an increase in expression of IGFBP-2 (Figure 3A, B). We did not detect any immunoreactivity in microglia in either the sham or MCAO groups (3C). Using an enzyme-linked immunosorbent assay (ELISA), we measured IGFBP-2 and IGF-I protein levels in the stroke brain. Sham and MCAO mice were sacrificed at 24 h or 72 h time points. The brains were removed and stained with TTC to visualize the penumbra. Tissue from the penumbra and stroke core was collected as shown in Figure 4. The olfactory bulb and cerebellum were also collected. There was no detectible difference in IGFBP-2 levels between the left and right hemisphere of sham mice after 24 h (Figure 5A) and 72 h (Figure 5C). IGFBP-2 levels increased after 24 h in stroke animals compared to sham animals, but there was no significant difference in olfactory bulb, penumbra and core IGFBP-2 levels between the contralateral and stroke hemispheres (Figure 5B). However, 72 h post-stroke, IGFBP-2 levels significantly increased in the stroke penumbra (5-fold) and core (3-fold) when compared to the contralateral hemisphere (Figure 5D). IGF-I levels in sham animals were not significantly different between the left and the right hemispheres for the brain regions analyzed. IGF-I was not detectable in the cortex at 24 h or 72 h (Figure 6A, C) or in the cerebellum at 72 h (Figure 6C). IGF-I levels increased 24 h post-stroke in the penumbra and core, but remained highest in the olfactory bulb (Figure 6B). Within 72 h, IGF-I levels were significantly higher at the stroke core when compared with contralateral hemisphere and also with the other brain regions (Figure 6D).

### Role of IGFBPs in intranasal transportation

**Figure 7:**
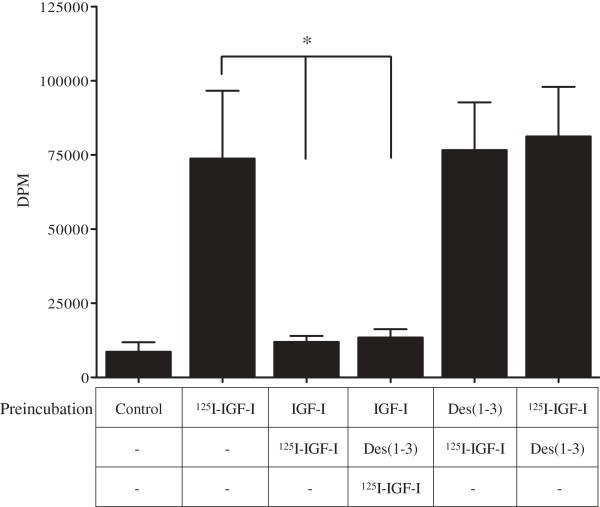
**Uptake of IGF-I is not mediated by IGF-IR.** Pre-incubation with a molar excess of Des(1–3)IGF-I (100 ng/ml), which does not bind to IGFBPs, does not block the uptake of ^125^I-IGF-I (20 ng/ml) into the brain. Pre-incubation with unlabeled IGF-I (20 ng/ml), which binds both to IGF-IR and IGFBPs, blocks ^125^I-IGF-I (20 ng/ml) uptake into the brain (*). Results are mean ± S.D. n = 6; *p < 0.05 by ANOVA ^125^I-IGF-I versus IGF-I + ^125^I-IGF-I and IGF-I + Des(1–3)IGF-I + ^125^I-IGF-I.

We investigated whether IGFBPs are required for the uptake of intranasally administered IGF-I into the CNS. Des(1–3)IGF-I, a potent IGF-I analog [28], can bind to the IGF-IR but cannot bind to IGFBPs [29]. We used the selective binding capability of Des(1–3)IGF-I to identify whether IGF-I uptake in the olfactory bulb requires IGF-IR or IGFBPs. We hypothesized that if IGF-I uptake into the olfactory bulb is mediated by IGF-IR, pre-incubation with Des(1–3)IGF-I would occupy all receptor-binding sites and significantly reduce the uptake of ^125^I-IGF-I into the brain. Des(1–3)IGF-I pre-incubation was not capable of blocking the uptake of subsequently applied ^125^I-IGF-I into the brain (Figure 7). Also, pre-incubation with ^125^I-IGF-I and immediate application of a molar excess of Des(1–3)IGF-I did not affect ^125^I-IGF-I uptake into the brain. However, pre-incubation with unlabeled IGF-I was capable of outcompeting ^125^I-IGF-I, in presence and absence of Des(1–3)IGF-I.

## Discussion

### IGFBP-2 & IGF-I protein levels in mouse brain

The 5-fold increase of the IGFBP-2 protein levels in the penumbra has two implications. It confirms the previous mRNA data and further demonstrates that the increase changes the distribution pattern of IGFBP-2 in control vs. stroke conditions (Figure 5). This region specific change in the protein distribution is particularly important as increased levels of IGFBP-2 in untreated animals in the penumbra indicates a possible neuroprotective role for endogenous IGFBP-2. This role is most likely associated with its regulation of IGF-I, and we show that IGF-I distribution in the brain changes in a pattern similar to IGFBP-2. IGF-I mRNA expression is detectable in many brain regions during embryogenesis, such as the spinal cord, midbrain, cerebral cortex, hippocampus, and olfactory bulb [13]. However, IGF-I transcription decreases significantly postnatally and reaches very low levels in the adult [30,31], with the exception of the olfactory bulb, where IGF-I expression persists at a high level throughout life [32]. We show that initially IGF-I is highly expressed in the olfactory bulb but is not detectable in other brain regions; however, within 72 h post-stroke it becomes increasingly abundant in the stroke core and penumbra (Figure 6). Such an increase in IGF-I levels in the stroke penumbra further supports the hypothesis that IGF-I is an integral part of the brain’s endogenous response to hypoxic-ischemic brain injury [33] and that IGFBP-2 also has a function in this response. Another region specific change of protein levels occurs in the stroke core where a significant increase of IGF-I and IGFBP-2 levels is observed. This might be due to IGFBP-2 being freed from its cell surface interactions with the necrotic cells. Even though the neurons in the stroke core are less likely to benefit from IGF-I, it might also be used to create a pool for IGF-I. Such a pool would be especially beneficial to the cells that are positioned in proximity to the necrotic (stroke core) border of the penumbra as it would provide these cells with a source for the neuroprotective factor IGF-I. Here, IGF-I can exert neuroprotection by initiating the Akt pathway and preventing neuronal apoptosis [34].

### Role of IGFBPs in intranasal transport

In order to investigate the role of IGFBPs in the uptake of IGF-I from intranasal cavity into the CNS, we used the selective binding capability of Des (1–3)IGF-I [29]. We hypothesized that if IGF-I uptake into the olfactory bulb is mediated by IGF-IR, pre-incubation or co-incubation with Des (1–3)IGF-I would compete for and occupy all the receptor-binding sites and significantly reduce the uptake of ^125^I-IGF-I (labeled IGF-I) into the brain. Our results indicate that such competition for the receptor does not affect labeled IGF-I uptake, entailing that IGF-I uptake is facilitated by another mechanism. This hypothesis is further supported by our finding that pre-incubation with unlabeled IGF-I, which binds to IGFBPs and IGF-IR, significantly reduced labeled IGF-I in the brain (Figure 7). Taken together, the results suggest the uptake of intranasal IGF-I is not receptor mediated and is most likely facilitated by IGFBPs. The significance of this finding is its potential link to drug delivery methods. Currently the prominent method is the systemic administration which relies on the unpredictable and unreliable permeabilization of the BBB [7]. However, relying on the breakdown of the BBB for access to the brain tissue is highly variable [35]. Therefore, exploration of alternative delivery methods, such as those involving binding protein mediated routes, might offer a more reliable and quick access to the brain tissue.

### An overview of IGFBP-2 in acute neuroprotection and long term recovery

IGFBP-2 has been widely studied in cancer research, where it was shown to be both inhibiting and stimulating for IGF-I linked tumor growth [36]. In the interpretation of IGFBPs role in hypoxic-ischemic injury a duality similar to the one observed in cancer research exists. There are several studies that demonstrated that IGFBPs antagonize IGF-I and limit its neuroprotective potential [37]. However, there are also studies that documented that IGFBP binding is essential for the IGF-I mediated neuroprotection [16]. Even though IGFBP-2 binding to IGF-I decreases IGF-I bioavailability, it also increases IGF-I half-life. This could be especially important if part of the brain’s endogenous response to hypoxic-ischemic injury involves transportation of IGF-I from other regions. The possibility for transportation is supported by our finding that IGFBP-2 and IGF-I distribution changes after stroke. While their levels are still high in the olfactory bulb, they become more abundant at the injury site. Whether this is due to local upregulation or transportation, presence of IGFBP-2 at the penumbra in itself is noteworthy. As described before [24] damage due to stroke is not uniform across the cortex. Stroke core can be defined as the areas where perfusion is completely absent during occlusion, resulting in necrotic tissue. Stroke penumbra, on the other hand, receives limited perfusion during occlusion. Cells in the penumbra have the potential to survive if treated, which in turn might improve the neurological and behavioral outcome post-stroke. Therefore, treatment studies utilizing IGF-I should focus on delivering the neuroprotectant to the stroke penumbra. It is at this point that IGFBP-2 might take the center stage by binding to IGF-I at the point of delivery or endogenous expression, protecting it from degradation, facilitating its transportation to the injury site and finally delivering IGF-I to its target. Furthermore, dominance of IGFBP-2 as the most abundant IGFBP in the olfactory bulb and its presence in the olfactory epithelia qualifies IGFBP-2 as a candidate for facilitating this uptake. This is supported by our finding that intranasally administered IGF-I most likely uses a currently uncharacterized IGFBP-mediated transport system to reach the brain. This also indicates that a similar pathway/system might exist for endogenous IGF-I transportation to the injury site as an endogenous response to brain injury.

Since our time points include the chronic phases of stroke, it is imperative to discuss the impact of the IGF system on the late remodeling of the injury site and surrounding tissue. We know from both human and animal studies [38,39], neuroprotective therapies for stroke are most effective within a 3–6 h time window after the initial insult [33] and that by 72 h, the size of the core stabilizes. Even if the IGF system still functions to stabilize the penumbra and delay and/or prevent neuronal death up to 24–48 h, there must be alternative explanations for their continuous upregulation up to 72 h after stroke. IGF-I has already been shown to be involved in neurovascular remodeling and neuroplasticity in penumbra and core at later time points [40,41]. We suggest that IGFBP-2 might also have an IGF-I dependent and independent role in such structural changes that occur in the chronic phases. This explanation would also support in situ hybridization studies where IGFBP-2 is found to localize with activated astrocytes and microglia [23,42]. Even though astrocyte and microglia proliferation, resulting in glial scarring, is known to be detrimental to neuronal survival, these cell types are also known to be associated with post-stroke angiogenesis and neurogenesis via expression of other proteins [43]. Hence their pro and anti-recovery roles need to be in balance [44]. IGF-I (neuroprotection) and IGFBP-2 (IGF-I regulation, angiogenesis, neurogenesis) might play a role in restoring such balance. Since tissue remodeling is a long term process [45], lasting increase in IGFBP-2 protein levels might be related to recovery mechanisms employed by various cell types in the CNS.

In the current paper we have provided data that intranasal IGF-I uptake is not IGF-IR mediated and showed that IGFBP-2 is present in the olfactory tissue, making it a likely candidate for transporting the intranasally administered IGF-I into the brain. However, no direct link has been established for IGFBP-2′s transport function of IGF-I into the stroke penumbra. Therefore, further competition experiments designed specifically towards IGFBP-2 are required to clarify IGFBP-2′s role. Similarly, IGF-I independent role of IGFBP-2 after stroke, needs to be further investigated using IGFBP-2 inhibition experiments. Such studies will clarify the acute function of IGFBP-2 in neuroprotection and also its long-term role in tissue recovery following a stroke event.

## Conclusions

The current results and the previous studies strongly suggest IGFBP-2′s role in hypoxic-ischemic injury needs to be explored further [46]. IGF-I is a promising neuroprotectant considered to be beneficial for not only stroke but a diverse array of neurological diseases such Amyotrophic Lateral Sclerosis (ALS) and traumatic brain injury [47,48]. Therefore, any insight into its mediation will allow us to take advantage of the brain’s existing neuroprotective and remodeling mechanisms. Establishing these mechanisms will in turn result in more effective treatments that incorporate improved administration methods and better dose regulation.

## Methods

All animal experiments were approved by the Institutional Animal Care and Use Committee of The University of Texas Health Science Center San Antonio and adhered to the National Institute of Health Guidelines.

### Cell culture

Primary cortical neurons and astrocytes were isolated from 17 day-old Sprague–Dawley rat embryos as described previously [49,50]. Briefly, the cerebral cortices were isolated and the meninges removed. The cortices were chopped into small pieces and then digested with trypsin-EDTA (0.125%) for 30 min at 37°C. The digested cells were mechanically dissociated by titration, filtered through a 40 μm cell strainer and collected by centrifugation (700 g, 2 min). For pure neuronal cultures, cells were re-suspended in serum-free Neurobasal Medium supplemented with B27, and plated at 5 × 10^5^ cells/cm^2^ onto poly-L-lysine coated glass coverslips. Cells were maintained at 37°C, 5% CO2, in a humidified environment, and allowed to mature for 17 days *in vitro* (DIV) [34]. For pure astrocyte cultures, cells were re-suspended in DMEM containing 10% fetal bovine serum, plated at 7,500 cells/cm^2^ onto tissue culture treated flasks and maintained at 37°C, 5% CO2 in a humidified environment until confluent (~14 DIV). Then, astrocytes were shaken overnight at 350 rpm to remove microglial cells and plated onto glass coverslips. The purity of each culture was tested using immunofluorescent techniques. Cells were labeled with a neuronal marker (microtubule associated protein 2 (MAP2), Sigma, M9942) and an astrocyte marker (glial fibrillary acidic protein (GFAP), Sigma, G6171). Neuronal cultures contained less than 3% GFAP-positive cells and astrocyte cultures did not contain any MAP2-positive cells.

### Oxygen–glucose deprivation and immunocytochemistry

The culture medium was replaced by a glucose-free Neurobasal-A media (Invitrogen), which was previously saturated with 1% O_2_. The cultures were then placed in an airtight incubation chamber (CBS Scientific) and flushed with a continuous influx of 1% O_2_ at a flow rate of 20 L/minute. The chamber was then sealed to maintain the gas composition and placed into an incubator at 37°C for 60 minutes. Afterwards the cultures were removed from the airtight hypoxic chamber and the glucose-free media was replaced with the pre-OGD conditioned medium. The cells were then maintained in normoxic conditions at 37°C for 24 h. Control cell cultures were not exposed to OGD. Cells were then rinsed in phosphate buffered saline (PBS), fixed in 4% paraformaldehyde for 10 minutes, and permeabilized with 0.5% Triton-X/PBS for 10 minutes. After blocking for 1 h at room temperature, the cells were incubated in primary antibodies (IGFBP-2, R&D Systems, AF797; MAP2, Sigma, M9942; GFAP, Sigma, G6171) and secondary antibodies (Alexa Fluors, 1:1000, Invitrogen) in blocking buffer. Coverslips were mounted onto slides with ProLong Gold Media (Invitrogen) and visualized with Zeiss AxioImage Olympus FV-1000 confocal microscope (Olympus America Inc.) and images captured with FluoView v. 5.0 software (Olympus America Inc.). We used an n = 3 for each experimental group.

### Transient middle cerebral artery occlusion (MCAO)

Adult male C57BL/6 mice weighing 20–25 g were anesthetized with 1.5-2% isoflurane. The animal’s body temperature was maintained at 37°C with a heating blanket and feedback system (Harvard Apparatus). Transient focal cerebral ischemia was induced by occlusion of the left MCA using the intraluminal filament model [51]. Reperfusion was performed by withdrawal of the filament 1 h after occlusion. Surface cerebral blood flow was monitored during MCAO by a laser doppler flowmeter (Perimed). Mice with remaining surface cerebral blood flow more than 20% of baseline were deemed to have unsuccessful MCAOs and were excluded from the experiment. Sham animals were subjected to the same surgical procedure as the stroke animals minus the occlusion of the MCA.

### Immunohistochemistry

Mice were transcardially perfused with normal saline followed by 4% paraformaldehyde 24 h post-stroke. Brains were harvested and immediately frozen in liquid nitrogen cooled isopentane. Coronal sections (25 μm) were cut with a crysostat (Leica) and fixed with acetone (10 m, -20°C). Sections were then permeabilized (0.1% Triton-X/PBS, 5 m, RT), blocked (5% Bovine Serum Albumin/PBS, 1.5 h, RT) and incubated in primary (IGFBP-2, R&D Systems, AF797; Neuronal Nuclei (NeuN), Millipore, MAB377; GFAP, Sigma, G6171; Iba-1, Wako, 019–19741) and secondary antibodies (Alexa Fluors, 1:500, Invitrogen) in blocking buffer. Coverslips were mounted onto slides with ProLong Gold Media (Invitrogen) and visualized with Zeiss AxioImage Olympus FV-1000 confocal microscope (Olympus America Inc.) and images captured with FluoView v. 5.0 software (Olympus America Inc.). We used an n = 3 for each experimental group.

### Tissue collection

Animals were perfused with saline solution. Olfactory epithelia, olfactory bulb, cortex (penumbra and core for the stroke group) and cerebellum were harvested using a dissecting microscope. In order to aid the collection of penumbra and core, 2 mm sections (Bregma +1 to −1) were stained with 2% 2,3,5-triphenyl tetrazolium chloride (TTC). Samples were sonicated (15%, 15 seconds) in lysis buffer (Cell Signaling) and centrifuged (14,000 rpm, 10 m, 4°C). Protein concentration of the supernatant was determined using bicinchoninic acid protein assay (Pierce). We used an n = 5 for each experimental group.

### Enzyme-linked immunosorbent assay (ELISA)

ELISA development kits (R&D Systems) were used to quantify IGF-I and IGFBP-2 levels in brain tissue, according to the manufacturer’s guidelines. A 96-well microplate was coated with the monoclonal capture antibody specific for IGFBP-2 (R&D Systems, MAB7971) or IGF-I (R&D Systems, MAB791). Wells were then incubated in blocking buffer, tissue homogenates (7.5 μg/well), standards, appropriate detection antibody (IGFBP-2 biotinylated antibody, R&D Systems, BAF797; IGF-I biotinylated antibody, R&D Systems, BAF791), and streptavidin-Horseradish Peroxidase (HRP) (Pierce). Following the streptavidin-HRP incubation, wells were covered with tetramethylbenzidine (TMB) substrate (Cell Signaling), and the HRP/TMB reaction was terminated with STOP Solution (Cell Signaling). The optical density was read at 450 nm using a spectrophotometer (Beckman Coulter Inc). Standard curves were used to interpolate the IGF-I and IGFBP-2 levels of the samples. Tissue homogenates were run in triplicates. We used an n = 5 for each experimental group.

### Intranasal administration

Des(1–3)IGF-I (GroPep) was diluted in 0.25% human albumin, 5.8 mg/ml sodium citrate, 5.8 mg/ml NaCl and 0.06 mg/ml citric acid. IGF-I and ^125^I-IGF-I were dissolved in 10 mM sodium succinate buffer and 140 mM NaCl (pH 6.2). A recently described method [3] for intranasal application was used. Treatment solutions were applied dropwise (2 μl/drop) to control mice over 12 m, alternating between each nare. The mouth and the alternate nare were closed during application to allow complete inhalation of the solution into the nasal cavity. Treatment solution incubation time was 20 m.

### Scintillation measurement

Whole brains (without the olfactory bulb) from mice were collected 20 minutes after the application of cytokines and homogenized in 1 ml buffer (50 mM Tris–HCl, pH 7.4) and mixed with 2 ml Microscint liquid scintillation cocktail (PerkinElmer) in a scintillation glass vial and measured in presence of I-125 Pico-Calibrator, #5080125, (PerkinElmer) in Topcount scintillation counter (PerkinElmer). We used an n = 6 for each experimental group.

### Data analysis

ELISA data was analyzed using Prism software (Graphpad). Graphs presented in the paper show mean and S.D. for n = 5 and the significance analysis. IGF-I and IGFBP-2 levels were analyzed by one-way analysis of variance (ANOVA, p < 0.05 was taken as statistically significant) followed by Tukey’s Multiple Comparison test. For scintillation data, differences in dpm levels were analyzed by one-way ANOVA (p < 0.05 was taken as statistically significant) for ^125^I-IGF-I versus IGF-I + ^125^I-IGF-I and IGF-I + Des(1–3)IGF-I + ^125^I-IGF-I. Results are shows as mean ± S.D.

## Competing interests

The author(s) declare that they have no competing interests.

## Authors’ contributions

LF and EI designed and performed experiments, analyzed data and drafted the manuscript. SS performed all animal surgeries. LHW and BBA carried out experiments and analyzed data. DFJ and MD conceived and oversaw the study, participated in experimental design and help draft the manuscript. All authors read and approved the final manuscript.
